# Application of Parameter Optimized Variational Mode Decomposition Method in Fault Feature Extraction of Rolling Bearing

**DOI:** 10.3390/e23050520

**Published:** 2021-04-24

**Authors:** Tao Liang, Hao Lu, Hexu Sun

**Affiliations:** 1 School of Artificial Intelligence and Data Science, Hebei University of Technology, Tianjin 300401, China; liangtao@hebut.edu.cn (T.L.); 18332901650@163.com (H.L.); 2 School of Electrical Engineering, Hebei University of Science and Technology, Shijiazhuang 050018, China

**Keywords:** rolling bearing, fault feature extraction, Variational Mode Decomposition, multi-island genetic algorithm, parameter optimization

## Abstract

The decomposition effect of variational mode decomposition (VMD) mainly depends on the choice of decomposition number K and penalty factor α. For the selection of two parameters, the empirical method and single objective optimization method are usually used, but the aforementioned methods often have limitations and cannot achieve the optimal effects. Therefore, a multi-objective multi-island genetic algorithm (MIGA) is proposed to optimize the parameters of VMD and apply it to feature extraction of bearing fault. First, the envelope entropy ($E_{e}$) can reflect the sparsity of the signal, and Renyi entropy ($R_{e}$) can reflect the energy aggregation degree of the time-frequency distribution of the signal. Therefore, $E_{e}$ and $R_{e}$ are selected as fitness functions, and the optimal solution of VMD parameters is obtained by the MIGA algorithm. Second, the improved VMD algorithm is used to decompose the bearing fault signal, and then two intrinsic mode functions (IMF) with the most fault information are selected by improved kurtosis and Holder coefficient for reconstruction. Finally, the envelope spectrum of the reconstructed signal is analyzed. The analysis of comparative experiments shows that the feature extraction method can extract bearing fault features more accurately, and the fault diagnosis model based on this method has higher accuracy.

## 1. Introduction

The failure of the wind turbine rolling bearing has a significant impact on the entire rotating machinery and even the operating state of the entire fan [1]. Due to the complex vibration transmission path of rolling bearing, it is very difficult to extract the fault information. Therefore, understanding how to effectively reduce the noise of the vibration signal and accurately extract fault features are key to early fault diagnosis of bearing [2,3].

In view of the above problems, scholars have put forward a variety of countermeasures. Empirical mode decomposition (EMD) can highlight the local characteristics of unstable signals and has good time-frequency aggregation ability [4]. However, the envelope estimation error of EMD will be amplified due to multiple recursive decompositions, which may be accompanied by modal aliasing, endpoint effect, pseudo pulse, and other phenomena [5]. To solve this problem, Wu et al. [6] proposed an ensemble empirical mode decomposition (EEMD), which effectively suppressed the phenomenon of mode aliasing. Gu et al. [7] used complete ensemble empirical mode decomposition with adaptive noise (CEEMDAN) to decompose the vibration signals, and then combined the signal quality index and singular value decomposition to extract local fault information of gearbox. Liu et al. [8] decomposed the vibration signal by local mean decomposition (LMD), and then adaptively learned the fault features combined with the SDAE model. However, these methods only reduce the mode aliasing phenomenon to a certain extent but do not completely eliminate it. Therefore, Dragomiretskiy et al. [9] proposed a variational mode decomposition algorithm. The bearing signal is processed into several sub-signals by the VMD algorithm to avoid the occurrence of mode aliasing. Jiang et al. [10] used the VMD algorithm to decompose the cylinder head vibration signal, and the obtained modal components to construct the characteristic matrix. Then, they calculated its singular value as the characteristic parameter, and then input it into the random forest classifier for diesel engine valve clearance fault diagnosis, and achieved good results. Qi et al. [11] used the VMD algorithm to process vibration signals into several IMFs and then combined with entropy value method and support vector machine classifier to complete bearing fault diagnosis.

However, the decomposition result of VMD largely depends on the choice of penalty factor α and decomposition number K. Wang et al. [12] proposed an improved VMD method based on the beetle antenna search (BAS) algorithm, which takes the kurtosis value of the intrinsic mode function as the fitness function in the search process, so as to optimize the parameters of the VMD algorithm. Gai et al. [13] optimized the parameters of VMD by the hybrid Gray Wolf algorithm and applied it to the extraction of bearing fault features. Yan et al. [14] optimized the parameters of VMD by a cuckoo search algorithm and applied it to fault detection of bearings.

As artificial intelligence technology progresses, bearing fault classification algorithms have become a hot topic for scholars. To learn the part-based representation of data and enhance sparseness, Zhang et al. [15] demonstrate the embedding of non-negativity constraints in the deep network. A state recognition method for the rolling bearing is proposed based on the deep autoencoder neural network with nonnegative constrains. In order to eliminate the distribution difference between training data and test data, Yu et al. [16] proposed an unsupervised domain adaptive fault diagnosis method based on a symmetric co-training framework. Given that it is difficult to obtain a large number of labeled data samples, Tao et al. [17] proposed an unsupervised rolling bearing fault diagnosis method based on short-time Fourier transform and categorical generative adversarial networks. The above bearing fault diagnosis methods belong to the category of neural network and deep learning. Although the neural network has a strong learning ability, it needs to set more parameters, and may converge too early to get the local optimal solution. The diagnosis accuracy of deep learning is relatively high, but its learning time is relatively long, and it needs a lot of labeled data. In order to simplify the classifier and detect the fault information of the signal, the envelope spectrum analysis of the fault signal is carried out.

On the basis of the above literature, this paper proposes a rolling bearing fault feature extraction method based on MIGA-VMD. Firstly, the bearing vibration signal is decomposed, and then the two IMFs with the most fault information are selected by the improved kurtosis and Holder coefficient for reconstruction, and the fault information is identified by envelope spectrum analysis. The contributions of the paper can be summarized as follows:

- A new fitness function is designed to optimize the parameters of VMD.
- The feature extraction method based on MIGA-VMD is studied through the actual data of rolling bearing.
- Compared with other feature extraction methods, the effectiveness of the proposed feature extraction method is verified.

The rest of the paper are organized as follows: Section 2 gives a brief introduction to the VMD method. In Section 3, the optimization algorithm and fitness function used to optimize VMD parameters are introduced, and the effectiveness of fitness function is proved. Section 4 introduces the improved kurtosis and Holder coefficient. The flow chart of the proposed method is given in Section 5, and the experimental analysis is carried out in Section 6. Finally, the conclusion of the paper is drawn in Section 7.

## 2. Brief Introduction of VMD

VMD is actually a method to solve variational problems. VMD can process a signal into K modal signals $u_{k}\left(k\in 1,2,\cdots ,K\right)$, the bandwidth of $u_{k}$ in the frequency domain has specific sparsity property [18]. The specific solving steps are as follows:

1. For each mode, the single side spectrum can be obtained by calculating the analytical signals related to each mode by Hilbert transform:
  (1) $$\left[\delta \left(t\right)+\frac{j}{\pi t}\right]*u_{k}\left(t\right).$$
2. An exponential term is added to modulate the spectrum of each mode to the corresponding baseband:
  (2) $$\left[\left(\delta \left(t\right)+\frac{j}{\pi t}\right)*u_{k}\left(t\right)\right]e^{-j\omega_{k}t}.$$
3. Each mode is near the center pulse frequency *ω_k_*, and the bandwidth of *ω_k_* is estimated by the *H*^1^ gaussian smoothness of the demodulated signal, i.e., the squared L2-norm of the gradient. In this way, we obtain a constrained variational problem [19]:
  (3) $$\left\{ \begin{array}{l} \underset{\left\{u_{k}\right\},\left\{\omega_{k}\right\}}{\min}\left\{\sum_{k=1}^{K}{\left\|\partial_{t}\left[\left(\delta \left(t\right)+\frac{j}{\pi t}\right)*u_{k}\left(t\right)\right]e^{-j\omega_{k}t}\right\|}_{2}^{2}\right\}\\ s.t.\sum_{k=1}^{K}u_{k}=x \end{array}\right.$$
  where x is the original signal, $u_{k}$ is the modal function, $\omega_{k}$ is the central frequency of each mode, δ(t) is the Dirichlet function, ∗ is the convolution operation.
4. In order to obtain the optimal solution of the constrained variational problem, the augmented Lagrange function is introduced:
  (4) $$\begin{array}{l} L\left(\left\{u_{k}\right\},\left\{\omega_{k}\right\},\lambda \right)=\\ \langle \lambda \left(t\right),x\left(t\right)-\sum_{k}u_{k}\left(t\right)\rangle +\\ {\left\|x\left(t\right)-\sum_{k}u_{k}\left(t\right)\right\|}_{2}^{2}+\alpha \sum_{k}{\left\|\partial_{t}\left[\left(\sigma \left(t\right)+\frac{j}{\pi t}\right)*u_{k}\left(t\right)\right]e^{-j\omega_{k}t}\right\|}_{2}^{2} \end{array}$$
  where α is the penalty factor and λ is the Lagrange factor.
5. In this way, the optimal solution problem of the constrained variational model can be transformed into the saddle point problem of the augmented Lagrange function, and the saddle point of the augmented Lagrange function can be obtained by using the alternating direction method of multipliers (ADMM). The expression of mode function is obtained as follows:
  (5) $${\hat{u}}_{k}^{n+1}\left(\omega \right)=\frac{\hat{x}\left(\omega \right)-\sum_{i=1,i\neq k}^{K}{\hat{u}}_{i}\left(\omega \right)+\frac{\hat{\lambda }\left(\omega \right)}{2}}{1+2\alpha {\left(\omega -\omega_{k}\right)}^{2}}$$
  where ${\hat{u}}_{k}^{n+1}\left(\omega \right)$ is the Wiener filter of the current residual $\hat{x}\left(\omega \right)-\sum_{i=1,i\neq k}^{k}{\hat{u}}_{i\left(\omega \right)}$, and the real part obtained by inverse Fourier transform of Wiener filter is $u_{k}\left(t\right)$. By transforming the center frequency problem into the frequency domain, the expression of center frequency can be obtained as follows:
  (6) $$\omega_{k}^{n+1}=\frac{\int_{0}^{\infty }\omega {\left|{\hat{u}}_{k}\left(\omega \right)\right|}^{2}d\omega }{\int_{0}^{\infty }{\left|{\hat{u}}_{k}\left(\omega \right)\right|}^{2}d\omega }$$
  where $\omega_{k}^{n+1}$ is the center frequency of the corresponding mode component.

The flow of the VMD algorithm is as follows.

1. Setting initial parameter $u_{k}^{1}$, $\omega_{k}^{1}$, $\lambda^{1}$, n as 0;
2. n=n+1, start the iteration cycle;
3. Update $u_{k}$ and $\omega_{k}$ according to expressions (5) and (6), where k cycles from 1 to K and K is the number of modes;
4. Update λ:
  (7) $$\lambda^{n+1}=\lambda^{n}+\tau \left(x-\sum_{k}u_{k}^{n+1}\right)$$
5. Given the decision accuracy ε>0, if the decision expression is satisfied:
  (8) $$\sum_{k}{\left\|{\hat{u}}_{k}^{n+1}-{\hat{u}}_{k}^{n}\right\|}_{2}^{2}/{\left\|{\hat{u}}_{k}^{n}\right\|}_{2}^{2}<\varepsilon$$
  then stop the iteration, otherwise return to step (2).

## 3. Multi-Objective Multi-Island Genetic Algorithm Optimizes the Parameters of VMD

According to reference [9], the VMD algorithm needs to determine the following parameters: Penalty parameter α, decomposition number K, updating parameters of Lagrange factor τ, center frequency initialization setting $\omega_{k}^{1}$, termination condition ε. In the process of VMD decomposition, the decomposition result of VMD mainly depends on the selection of penalty parameter α and decomposition number K, and the other parameters are set according to experience, that is, τ=0, $\omega_{k}^{1}=0$, ε=1e−7. Under specific α and K, each IMF component has limited bandwidth. The size of bandwidth depends on the setting of penalty parameter α. The smaller α is, the smaller the bandwidth is; the larger α is, the larger the bandwidth is. In addition, the choice of decomposition number K is more important. If K is too small, it will lead to mode aliasing; if K is too large, it will lead to the generation of useless components. Therefore, it is necessary to set the appropriate α and K through the optimization algorithm. In this paper, the selection range of α is [50,3500], and the selection range of K is [2,12].

The PSO algorithm, introduced by Kennedy and Eberhart [20,21], is a population-based stochastic approach for solving continuous and discrete optimization problems. Its advantage is that there are not too many complex parameters to adjust, and it is easy to achieve, so some scholars optimize the parameters of VMD through a particle swarm optimization algorithm [22]. However, the PSO algorithm has the disadvantage of premature convergence. The genetic algorithm simulates the process of biological evolution, including selection, crossover, mutation, and other operations, relying on continuous evolution to get the optimal solution [23]. Its advantage is that the search starts from the group and has potential parallelism. The genetic algorithm is also easy to converge in advance, which affects its own optimization ability. In order to prevent premature convergence, a multi-island genetic algorithm is proposed. The algorithm adds many “islands” on the basis of the genetic algorithm, and there will be some individuals on each island. It is assumed that individuals can migrate between each island, and those with migration ability are excellent individuals, which can help the algorithm avoid premature convergence [24]. The flow of MIGA is shown in Algorithm 1.
**Algorithm 1** Multi-Island Genetic Algorithm1: Randomly generate N individuals as the initial population $P_{0}$.2: The population $P_{0}$ is divided into some “islands”.3: The fitness values of each individual in the “island” were obtained.4: Perform operations such as selection, crossover, and mutation on the island.5: If the migration condition is satisfied, the individual will migrate from the current island to other islands.6: If the current number of iterations does not reach the set value, step 3 will be returned, otherwise step 7 will be executed.7: The optimal solution is the one with the least fitness among all individuals.

When using a multi-objective multi-island genetic algorithm to optimize VMD parameters, the key is to select appropriate fitness functions. The concept of envelope entropy $E_{e}$ is proposed in the literature [25]. The bearing signal is decomposed into multiple IMF components by VMD, and the $E_{e}$ value of the component can represent the sparsity of the component signal. The larger the $E_{e}$ value is, the smaller the sparsity of the IMF component is and the more noises it contains; the smaller the $E_{e}$ value is, the greater the sparsity of the IMF component is and the more periodic shocks it contains. Therefore, the fitness function is constructed as follows:

(9) $$\min F_{1}=\min E_{e}$$

where $E_{e}$ is calculated as follows:

(10) $$\left\{ \begin{array}{l} E_{e}=-\sum_{j=1}^{N}e_{j}\log e_{j}\\ e_{i}=a\left(j\right)/\sum_{j=1}^{N}a\left(j\right) \end{array}\right.$$

where j=1,2,3,…,N, a(j) is obtained by Hilbert demodulation of the original signal.

In order to prove that $E_{e}$ can be used as fitness function, we construct a simulation signal of faulty bearing, and the expression is as follows:

(11) $$\left\{ \begin{array}{l} f(t)=y_{0}e^{-2\pi f_{n}\xi t}\sin\left(2\pi f_{n}\sqrt{1-\xi^{2}}t\right)\\ c\left(t\right)=\sum_{\tau }f(t-\tau )+n\left(t\right) \end{array}\right.$$

where t is the sampling time, τ=0, 0.02, 0.04,0.06…, f(t) stands for the single impulse response, n(t) represents Gaussian white noise, and the noise intensity is determined by the standard deviation, and c(t) is a sum of time-shifted impulse responses with noise. Here, we set $y_{0}=3$, $f_{n}=3000\;\mathrm{Hz}$, ξ=0.09. In addition, sampling frequency $f_{s}=20\;\mathrm{kHz}$, sampling number N=4096.

**Figure 1:**
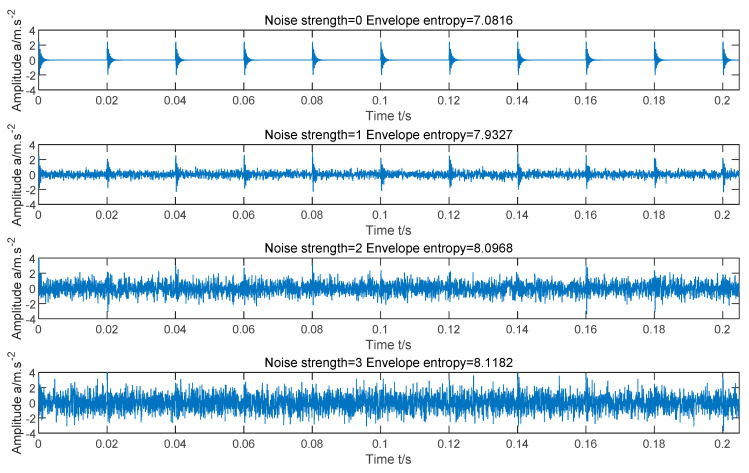
Time-domain waveforms of simulated signals under different noise intensities.

Figure 1 shows the vibration waveforms of the faulty bearing simulation signal when there is no noise, the noise intensity is 1, the noise intensity is 2, and the noise intensity is 3. It can be seen from the figure that with the increase of noise intensity, the background noise will gradually submerge the fault impact. By comparing the four images, it can be found that the greater the noise intensity of the signal is, the more blurred the periodic pulse becomes, the weaker the sparsity of the signal is, and the greater the $E_{e}$ value is; The less noise the signal contains, the more significant the periodic pulse becomes, the stronger the sparsity of the signal is, and the smaller the $E_{e}$ value is. Therefore, the smaller the $E_{e}$ value of the signal, the more obvious the fault impact of the signal, and $E_{e}$ can be used as a fitness function.

Renyi entropy $R_{e}$ further expands the concept of information entropy, which can reflect the energy aggregation of signal in time-frequency distribution and the complexity of signal [26]. Compared with information entropy, Renyi entropy is more sensitive to the change of signal and easier to identify the small change of signal [27]. For a fault signal, if the noise is smaller, the main frequency will be more concentrated, the energy aggregation will be better, and the $R_{e}$ value of the signal will be smaller; If the noise is bigger, the complexity will be bigger, the energy aggregation will be worse, and the $R_{e}$ value of the signal will be bigger. In other words, the smaller the $R_{e}$ value is, the less noise the signal contains and the better the energy aggregation of the signal is. Therefore, the fitness function is constructed as follows:

(12) $$\min F_{2}=\min R_{e}$$

where $R_{e}$ is defined as:

(13) $$R_{e}(X)=\frac{1}{1-\alpha }\sum_{i=1}^{n}\log p_{k}^{\alpha }$$

where α≥0, indicating the order of $R_{e}$; $p_{k}$ is the probability density of $X=x_{k}$.

**Figure 2:**
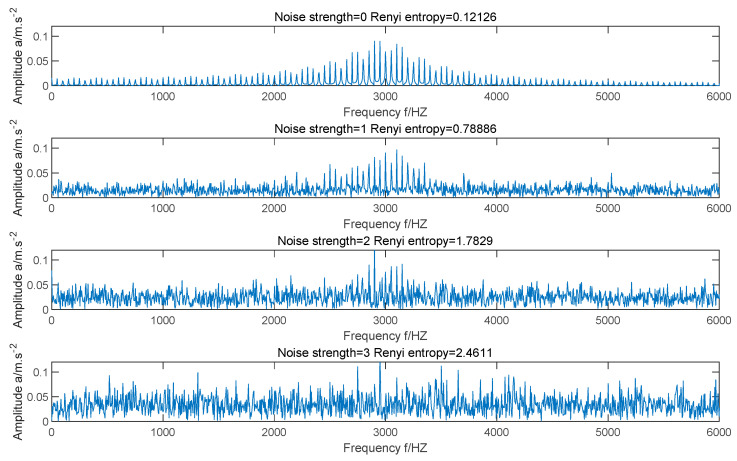
Frequency domain waveforms of simulated signals under different noise intensities.

In order to prove that $R_{e}$ can be used as fitness function, we still use Equation (11) as simulation signal of faulty bearing. Figure 2 shows the frequency-domain waveforms of the simulation signal when there is no noise, the noise intensity is 1, the noise intensity is 2, and the noise intensity is 3. It can be seen from the figure that with the increase of noise intensity, the main frequency in the signal is gradually submerged by the background noise. By comparing the four images, we can find that the less noise the signal contains, the more concentrated the main frequency is, the better the energy aggregation is, and the smaller the $R_{e}$ value is; The larger the noise intensity of the signal is, the more scattered the main frequency is, the worse the energy aggregation is, and the greater the $R_{e}$ value is. Therefore, the smaller the $R_{e}$ value of the signal, the more it contains the main fault information, and $R_{e}$ can be used as the fitness function.

Due to the influence of parameters K and α, each IMF has its own $E_{e}$ value and $R_{e}$ value. The smallest one of K $E_{e}$ values is selected as the local minimum $E_{e}$, and the smallest one of K $R_{e}$ values is selected as the local minimum $R_{e}$, the components corresponding to these two minimum entropy values contain rich feature information [28]. The average value of local minimum $E_{e}$ and local minimum $R_{e}$ is taken as fitness function to search the most suitable parameters K and α. Therefore, the final fitness function is as follows:

(14) $$\min F=\frac{1}{2}\left(\min E_{e}+\min R_{e}\right)$$

## 4. Brief Introduction of Improved Kurtosis and Holder Coefficient

Bearing fault signals are decomposed into some IMFs by MIGA, and only some of these IMFs contain bearing fault information. Therefore, screening the most effective IMF is more conducive to fault feature extraction, but this has been ignored in most studies. The holder coefficient can measure the correlation between signals, and the larger the holder coefficient is, the more significant the correlation is [29]. Kurtosis is easy to sense local fault impact, so it is widely used to screen IMF containing bearing fault information, but the interference pulse affects the effect of kurtosis index [30]. Therefore, it is necessary to eliminate the influence of some extreme values when calculating kurtosis. The calculation method of improved kurtosis is as follows:

For a vibration signal x(t)=[x(1),x(2),…,x(N)], whose absolute value vector is:

(15) y(t)=|x(t)|,t=1,2,…,N

The extremum of signal x(t) corresponds to the maximum point of y(t), the kernel density estimation method is used to calculate the probability density function of y(t), as follows:

(16) $$F(y)=\frac{1}{Nd\sqrt{2\pi }}\sum_{t=1}^{N}\exp\left\{-\frac{{\left(y-\left|x(t)\right|\right)}^{2}}{2d^{2}}\right\}$$

If F(y) is greater than α(0≤α≤1), the minimum value of y can be expressed as:

(17) $$y_{low}=\min\left\{y:F(y)=\int_{u\leq y}f(s)ds\geq \alpha \right\}$$

where α is a critical probability and F(y) is the probability distribution function of signal y(t). If α tends to 1, then $y_{low}$ can be regarded as the boundary-value extreme point. If the absolute value y(t) of the sample point x(t) is greater than $y_{low}$, then the sample point x(t) is regarded as the extreme point.

In order to reduce the influence of these abnormal extreme points on kurtosis, we use the linear interpolation algorithm [31] to replace these abnormal extreme points. Then, we calculate the kurtosis of the adjusted signal $x_{adj}$:

(18) $$K_{adj}=\frac{\sum_{t=1}^{N}{\left(x_{adj}(t)\right)}^{4}}{{\left(\sum_{t=1}^{N}{\left(x_{adj}(t)\right)}^{2}\right)}^{2}}-3$$

For two signal samples $x\left(t\right)=\left[x_{1},x_{2},\cdots ,x_{n}\right]$ and $y\left(t\right)=\left[y_{1},y_{2},\cdots ,y_{n}\right]$ with n values, the Holder coefficient is defined as:

(19) $$H=\frac{\sum_{i=1}^{N}x_{i}y_{i}}{{\left(\sum_{i=1}^{N}x^{p}\right)}^{1/p}\cdot {\left(\sum_{i=1}^{N}y^{q}\right)}^{1/q}}$$

where 1/p+1/q=1 and p, q>1; $0\leq H_{c}\leq 1$.

In this paper, improved kurtosis and Holder coefficient are used to select the most effective IMF for fault feature extraction.

## 5. Fault Feature Extraction Model Based on MIGA-VMD

**Figure 3:**
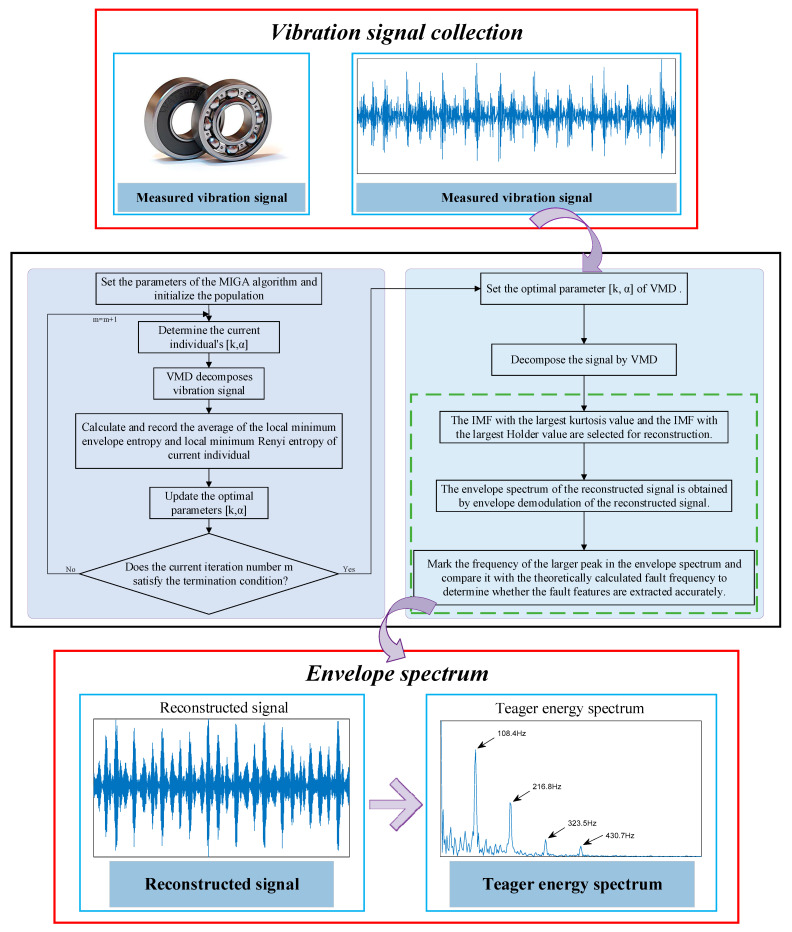
Flow chart of the proposed method.

In general, the proposed fault feature extraction model is shown in Figure 3, and the implementation process is as follows:

Step 1: Taking the average value of local minimum $E_{e}$ and local minimum $R_{e}$ as the fitness function, using the MIGA algorithm to search the most suitable VMD parameters K and α.

Step 2: The VMD with optimized parameters is used to process the vibration signals of different states, and K IMFs are obtained, respectively.

Step 3: Calculate the improved kurtosis of each IMF; calculate Holder coefficients between each IMF and the original signal.

Step 4: The IMF with the largest kurtosis value and the IMF with the largest Holder value are selected for reconstruction.

Step 5: The envelope spectrum of the reconstructed signal is obtained by Teager energy operator envelope demodulation.

Step 6: The frequency of the larger peak value is marked in the envelope spectrum, which is compared with the bearing fault frequency obtained by theoretical calculation, so as to judge whether the fault feature is extracted accurately.

## 6. Experimental Study

### 6.1. Introduction of Experiment

**Figure 4:**
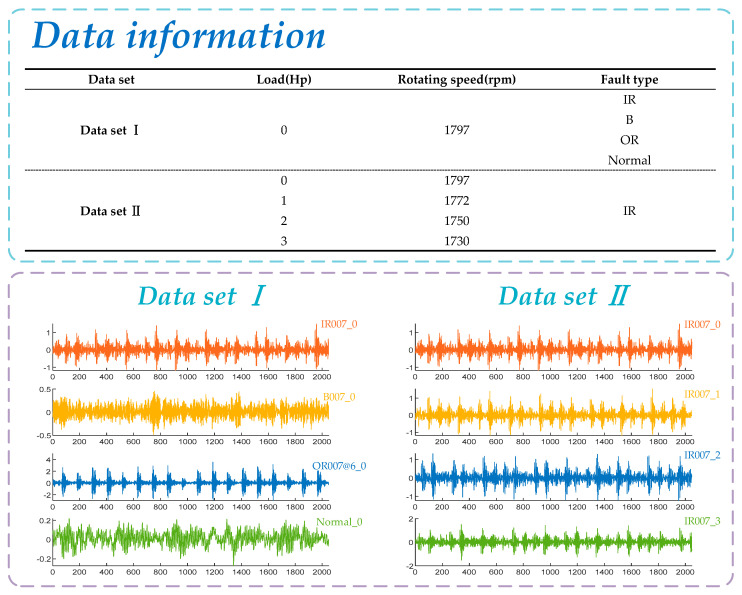
Information on bearing vibration data.

The experimental data are from Case Western Reserve University (CWRU) Bearing Data Center [32]. The experimental center processes single point damage on SKF6205 bearing by EDM technology and uses an acceleration sensor to measure the bearing vibration signal. Other parameters of bearing are shown in Table 1. The data contains multiple sets of data under different conditions. Here, the bearing drive ends signals with the sampling frequency of 12,000 Hz and load of 0 Hp are selected for simulation verification, including inner race fault signal, ball fault signal, outer race fault signal, and normal signal. The sample length of this experiment is set to 2048, and the specific data information is shown in Figure 4.

### 6.2. Comparison and Analysis of the Experiment

The load is 0 Hp, the corresponding motor speed is 1797 rpm, so the motor frequency $f_{0}$ is 29.95 Hz. According to the empirical formula (21)–(23) of bearing fault frequency, the theoretical inner race fault frequency $f_{1}$, ball fault frequency $f_{2}$, and outer race fault frequency $f_{3}$ are 162.19 Hz, 141.16 Hz, and 107.36 Hz, respectively.

(20) $$f_{0}=29.95\mathrm{Hz}$$

(21) $$f_{1}=\frac{Z}{2}\times \left(1+\frac{d}{D}\times \cos\alpha \right)\times \frac{N}{60}$$

(22) $$f_{2}=\frac{1}{2}\times \frac{D}{d}\times \left(1-{\left(\frac{d}{D}\right)}^{2}\times \cos^{2}\alpha \right)\times \frac{N}{60}$$

(23) $$f_{3}=\frac{Z}{2}\times \left(1-\frac{d}{D}\times \cos\alpha \right)\times \frac{N}{60}$$

**Figure 5:**
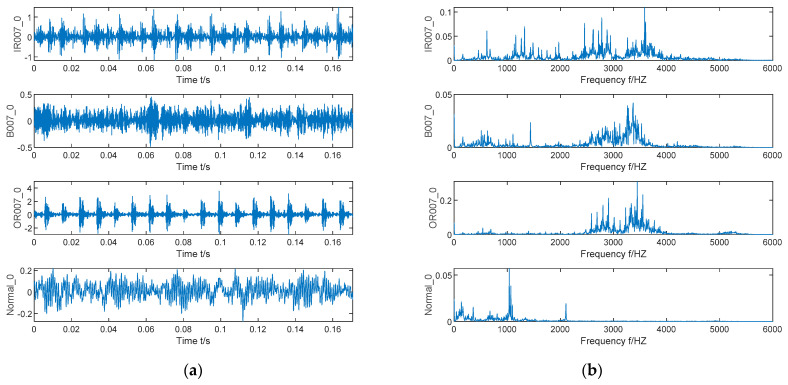
Time-domain diagram and frequency domain diagram of vibration signals of inner race fault, ball fault, outer race fault, and normal state. (**a**) Time-domain diagram; (**b**) frequency domain diagram.

The time domain and frequency domain waveforms of bearing vibration signals under inner race fault, ball fault, outer race fault, and normal state are shown in Figure 5. Due to the influence of noise interference, it is difficult to observe the obvious fault frequency from Figure 5. Therefore, in the follow-up experiments, the VMD algorithm is used to process the bearing vibration signal.

**Figure 6:**
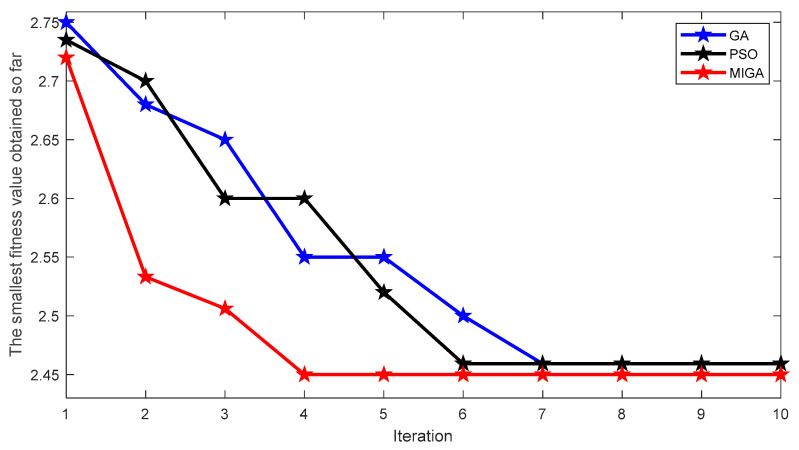
Performance comparison of PSO algorithm, GA algorithm, and MIGA algorithm.

Prior to using the VMD algorithm to process the bearing signal, it is necessary to use optimization algorithm to determine the most appropriate parameters K and α. In order to prove the convergence and optimization performance of MIGA, different algorithms are used to optimize the VMD parameters of inner race fault signal, including PSO algorithm, GA algorithm, and MIGA algorithm. In the process of testing, Equation (14) is used as the fitness function of the three algorithms. Figure 6 shows the change process of fitness values of the three algorithms in the optimization process.

It can be seen from Figure 6 that in this test, PSO algorithm converges faster than GA algorithm, but both PSO algorithm and GA algorithm converge in advance. The MIGA algorithm used in this paper not only has better convergence speed than PSO algorithm and GA algorithm, but also avoids falling into a local optimum and has better optimization ability.

**Figure 7:**
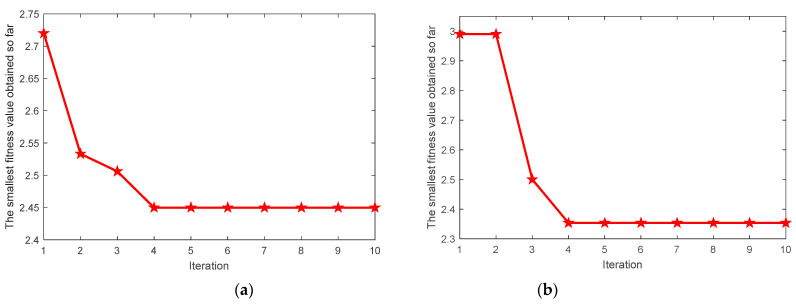

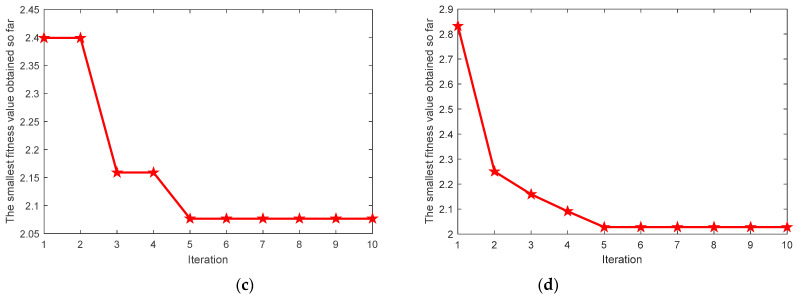
The MIGA convergence curve for VMD parameter optimization. (**a**) Inner race fault; (**b**) ball fault; (**c**) outer race fault; (**d**) normal.

Taking the average value of local minimum $E_{e}$ and local minimum $R_{e}$ as the fitness function, the most suitable parameters K and α are searched by the MIGA algorithm. Figure 7 shows the change process of fitness values of four types of signals with the increase of iterations in the optimization process. Finally, the VMD optimal parameter combinations $\left[K_{0},\alpha_{0}\right]$ of four types of signals are found as [11,350], [9,3241], [7,3497], [11,3498]. According to the optimal parameter combination $\left[K_{0},\alpha_{0}\right]$, the VMD parameters of each state signal are set and the signal is decomposed by VMD.

**Figure 8:**
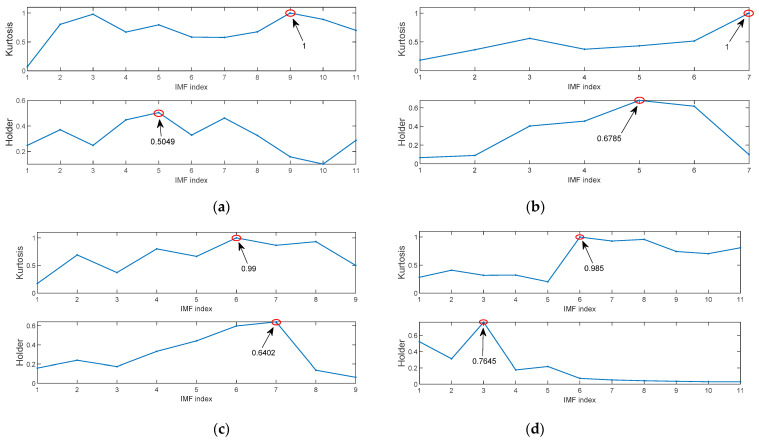
The improved kurtosis values and Holder coefficient values of each IMF after MIGA-VMD decomposition of four different state signals. (**a**) Inner race fault; (**b**) Ball fault; (**c**) Outer race fault; (**d**) Normal.

The improved kurtosis and Holder coefficient can evaluate the degree of IMF containing fault information. The improved kurtosis values and Holder coefficient values of each IMF after four types of signals are decomposed by the MIGA-VMD algorithm are shown in Figure 8.

**Figure 9:**
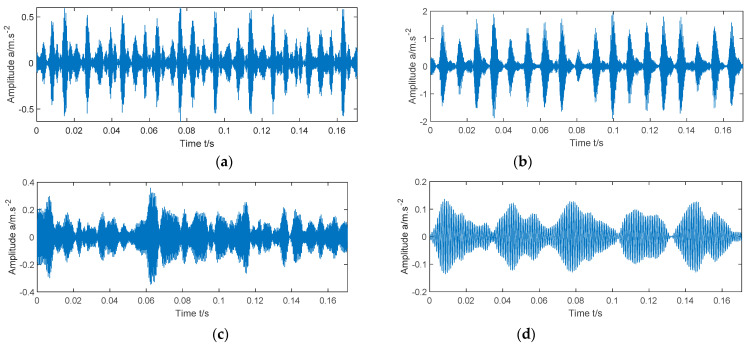
Time-domain diagrams of reconstructed signals of four different state signals. (**a**) Inner race fault; (**b**) Ball fault; (**c**) outer race fault; (**d**) normal.

**Figure 10:**
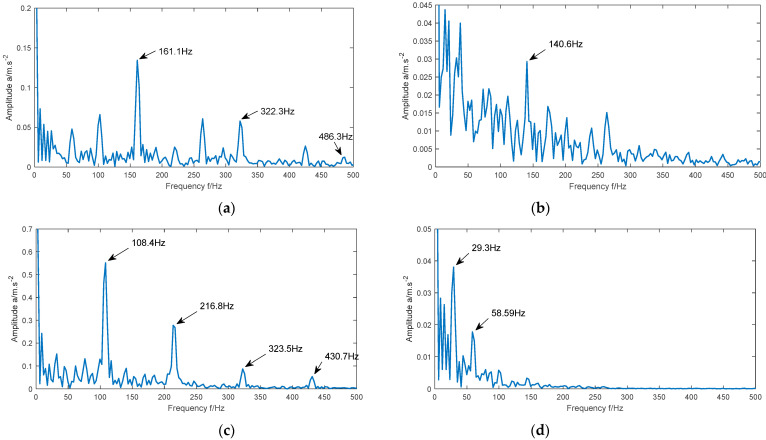
Envelope spectrum of the reconstructed signal of four different state signals. (**a**) Inner race fault; (**b**) ball fault; (**c**) outer race fault; (**d**) normal.

The IMF with the largest kurtosis value and the IMF with the largest Holder value are selected for reconstruction. The time-domain waveform and envelope spectrum of the reconstructed signals of the four types of signals are shown in Figure 9 and Figure 10, respectively.

It can be seen from Figure 10a that the maximum peak value appears in the figure when the frequency is 161.1 Hz, and the obvious peak value also appears in the figure when the frequency is 322.3 Hz and 486.3 Hz. These three frequencies are close to the theoretical fault frequencies $f_{1}$, $2f_{1}$, and $3f_{1}$, respectively. It can be seen from Figure 10b that the maximum peak value appears in the figure when the frequency is 140.6 Hz, which is close to the theoretical fault frequency $f_{2}$. As can be seen from Figure 10c, when the frequencies are 108.4 Hz, 216.8 Hz, 323.5 Hz, and 430.7 Hz, obvious peaks appear in the figure. The four frequencies are close to the theoretical fault frequencies $f_{3}$, $2f_{3}$, $3f_{3}$, and $4f_{3}$ respectively. As can be seen from Figure 10d, when the frequencies are 29.3 Hz and 58.59 Hz, obvious peaks appear in the figure. These two frequencies are close to the theoretical motor frequencies $f_{0}$ and $2f_{0}$. Due to the fault frequency of different fault signals is different, it can be determined that the method in this paper can accurately extract the fault features of the bearing vibration signal.

In order to further verify the effectiveness of the proposed method, the inner race fault signals of 1797 rpm, 1772 rpm, 1750 rpm, and 1730 rpm are tested. According to the formula (21), the inner race fault frequencies $f_{rpm1}$, $f_{rpm2}$, $f_{rpm3}$, and $f_{rpm4}$ under four rotational speed conditions are 162.19 Hz, 159.93 Hz, 157.94 Hz, and 156.14 Hz.

**Figure 11:**
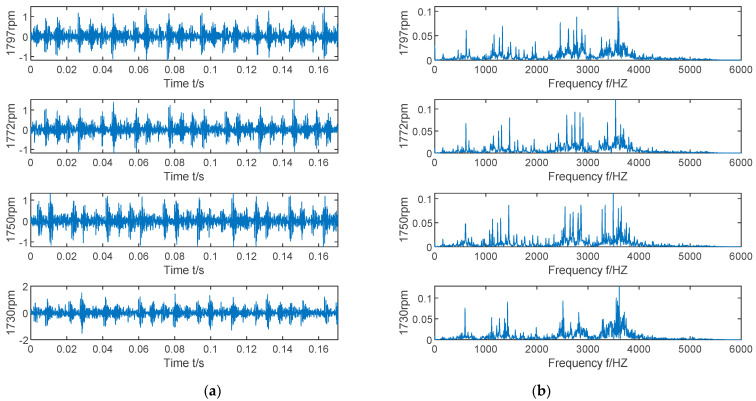
Time-domain diagram and frequency domain diagram of vibration signals of 1797 rpm, 1772 rpm, 1750 rpm, and 1730 rpm. (**a**) Time-domain diagram; (**b**) frequency domain diagram.

The time domain waveform and frequency domain waveform of bearing vibration signals under four rotational speed conditions are shown in Figure 11.

**Figure 12:**
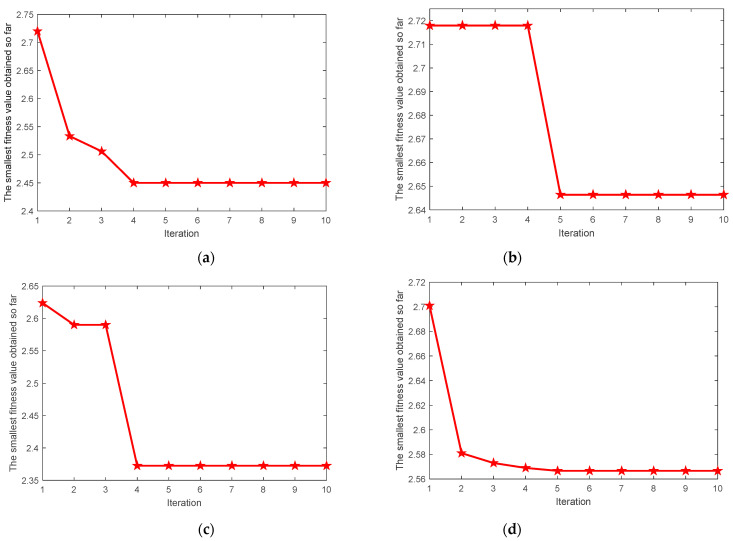
The MIGA convergence curve for VMD parameter optimization. (**a**) 1797 rpm; (**b**) 1772 rpm; (**c**) 1750 rpm; (**d**) 1730 rpm.

Taking the average value of local minimum $E_{e}$ and local minimum $R_{e}$ as the fitness function, the most suitable parameters K and α are searched by the MIGA algorithm. Figure 12 shows the change process of fitness values of signals under four rotational speed conditions with the increase of iterations in the optimization process. Finally, the VMD optimal parameter combinations $\left[K_{0},\alpha_{0}\right]$ of signals under four rotational speed conditions are found as [11,350], [10,3236], [11,750], [12,501]. According to the optimal parameter combination $\left[K_{0},\alpha_{0}\right]$, the VMD parameters of signals under four rotational speed conditions are set and the signal is decomposed by VMD.

**Figure 13:**
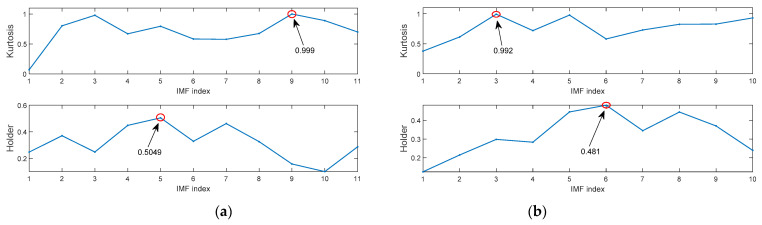

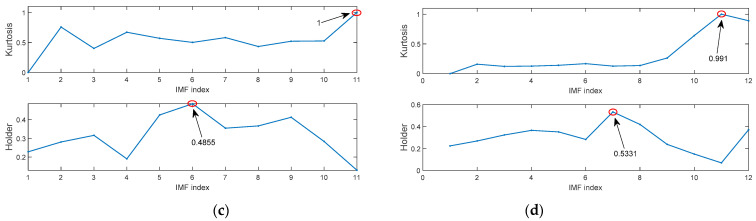
The improved kurtosis values and Holder coefficient values of each IMF after MIGA-VMD decomposition of four different state signals. (**a**) 1797 rpm; (**b**) 1772 rpm; (**c**) 1750 rpm; (**d**) 1730 rpm.

The improved kurtosis values and Holder coefficient values of each IMF after signals under four rotational speed conditions are decomposed by the MIGA-VMD algorithm are shown in Figure 13.

**Figure 14:**
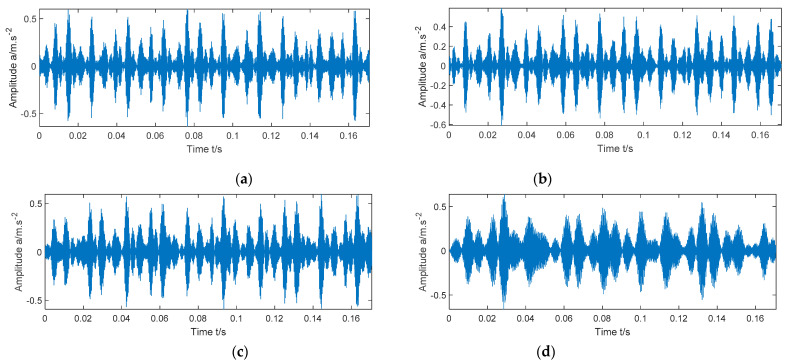
Time-domain diagrams of reconstructed signals of signals under four rotational speed conditions. (**a**) 1797 rpm; (**b**) 1772 rpm; (**c**) 1750 rpm; (**d**) 1730 rpm.

**Figure 15:**
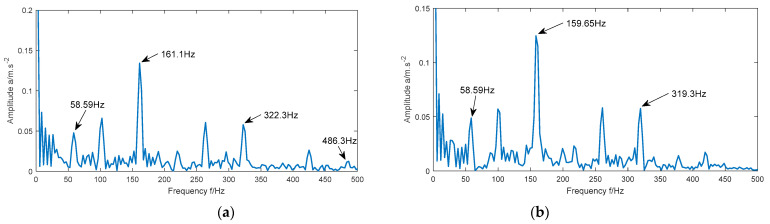

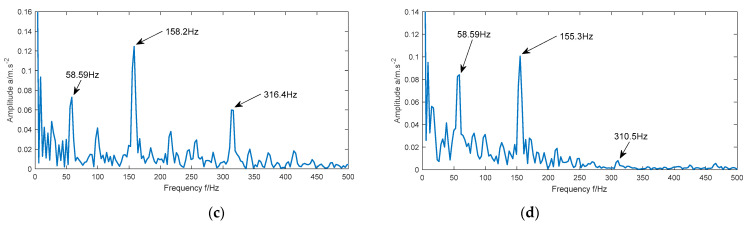
Envelope spectrum of the reconstructed signal of signals under four rotational speed conditions. (**a**) 1797 rpm; (**b**) 1772 rpm; (**c**) 1750 rpm; (**d**) 1730 rpm.

The IMF with the largest kurtosis value and the IMF with the largest Holder value are selected for reconstruction. The time-domain waveform and envelope spectrum of the reconstructed signals of signals under four rotational speed conditions are shown in Figure 14, and Figure 15, respectively.

It can be seen from Figure 15a that the maximum peak value appears in the figure when the frequency is 161.1 Hz, and the obvious peak value also appears in the figure when the frequency is 322.3 Hz and 486.3 Hz. These three frequencies are close to the theoretical fault frequencies $f_{rpm1}$, $2f_{rpm1}$, and $3f_{rpm1}$, respectively. Figure 15b shows that when the frequencies are 159.65 Hz and 319.3 Hz, obvious peaks appear in the figure. These two frequencies are close to the theoretical fault frequencies $f_{rpm2}$ and $2f_{rpm2}$. As can be seen from Figure 15c, when the frequencies are 158.2 Hz and 316.4 Hz, obvious peaks appear in the figure. These two frequencies are close to the theoretical fault frequencies $f_{rpm3}$ and $2f_{rpm3}$. As can be seen from Figure 15d, when the frequencies are 155.3 Hz and 310.5 Hz, obvious peaks appear in the figure. These two frequencies are close to the theoretical fault frequencies $f_{rpm4}$ and $2f_{rpm4}$. In addition, the peak value appears when the frequency is 58.59 Hz in the four figures, which is the motor frequency. As the fault frequency of signals under different rotational speed conditions is different, it can be determined that the method, in this paper, can accurately extract the fault features of the bearing vibration signal.

### 6.3. Comparison of Feature Extraction Methods

**Figure 16:**
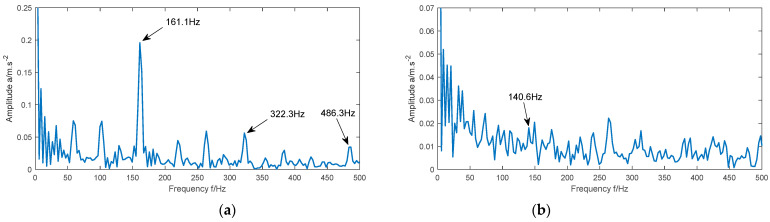

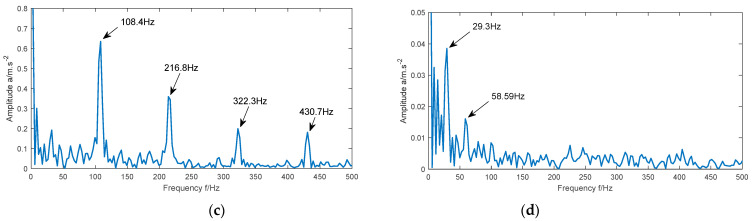
The envelope spectrum of the reconstructed signal of the four different state signals processed by the CEEMDAN algorithm. (**a**) Inner race fault; (**b**) ball fault; (**c**) outer race fault; (**d**) normal.

**Figure 17:**
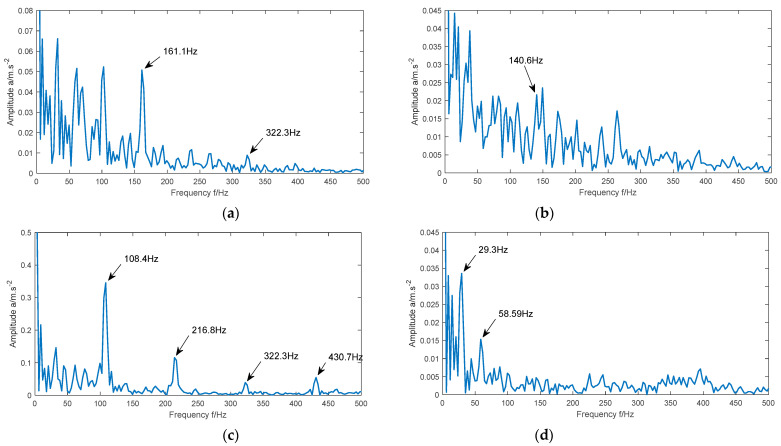
The envelope spectrum of the reconstructed signal of the four different state signals processed by the traditional VMD algorithm. (**a**) Inner race fault; (**b**) ball fault; (**c**) outer race fault; (**d**) normal.

In order to verify the superiority of the MIGA-VMD algorithm, the CEEMDAN algorithm and traditional VMD algorithm are used to replace the MIGA-VMD algorithm. Figure 16 and Figure 17 are the envelope spectrum of reconstructed signals of four types of signals processed by the CEEMDAN algorithm and traditional VMD algorithm respectively. In the traditional VMD algorithm, K and α are set to [28]: K=6, α=2000.

It can be seen from Figure 16a that the maximum peak value appears in the figure when the frequency is 161.1 Hz, and the obvious peak value also appears in the figure when the frequency is 322.3 Hz and 486.3 Hz. Although these three frequencies are close to the theoretical fault frequencies $f_{1}$, $2f_{1}$, and $3f_{1}$ respectively, there are many interference frequencies in the figure, which may affect the accuracy of fault feature extraction. As shown in Figure 16b, although there is a peak at 140.6 Hz, the frequency is submerged in other noise frequencies, so it is difficult to obtain it accurately. As shown in Figure 16c, there are seven obvious peaks in the figure, and their frequencies are close to the theoretical fault frequencies $f_{3}$, $2f_{3}$, $3f_{3}$, and $4f_{3}$ respectively. As shown in Figure 16d, when the frequencies are 29.3 Hz and 58.59 Hz, obvious peaks appear in the figure. Although these two frequencies are close to the theoretical motor frequencies $f_{0}$ and $2f_{0}$, there are also many interference frequencies in the figure. In Figure 17, similar situations also appear, such as the fault frequency is not obvious, a large number of noise frequency are doped, and even the fault frequency is submerged by the noise frequency. 

To sum up, the results processed by the MIGA-VMD algorithm show obvious fault information, and the fault frequency is clearly visible. Although, the CEEMDAN algorithm and traditional VMD algorithm can also extract part of the feature information, it is mixed with more noise interference, resulting in the feature frequency is not clear or completely submerged in the noise. Therefore, for the fault feature extraction of the rolling bearing vibration signal, the performance of the MIGA-VMD algorithm is better than that of the CEEMDAN algorithm and traditional VMD algorithm.

### 6.4. Performance of Bearing Fault Diagnosis

In order to further prove the effectiveness of the proposed feature extraction method, this method is combined with the classifiers in three literatures for bearing fault diagnosis, and the diagnosis accuracy is compared with the original method, as shown in Table 2.

In Table 2, literature [13] decomposes the bearing signal by EMD, and then calculates the singular value of the matrix composed of IMFs as the feature vector, which is input into the DBN classifier. The accuracy of the diagnosis result is 93.55%. In this paper, MIGA-VMD method is used to replace EMD method, and then combined with SVD and DBN. The diagnostic accuracy is 95.1%. In literature [26], the bearing signal is decomposed by EEMD, and the Renyi entropy of each IMF is calculated to form the feature vector, and then reduced dimension by PCA. Finally, it is input into PNN classifier, the diagnostic accuracy is 91.7%. In this paper, MIGA-VMD method is used to replace EEMD method, and then combined with Renyi entropy, PCA, and PNN. The diagnostic accuracy is 93.9%. In literature [32], the original signal of bearing is directly input into 1-DCNN classifier, and the accuracy of diagnosis result is 99.34%. In this paper, the original signal is processed by MIGA-VMD, and the two IMF which contain the most fault information are reconstructed, and then it is input into the 1-DCNN classifier. The accuracy of the diagnosis result is 99.7%. To sum up, the combination of the proposed feature extraction method and the classifiers in the literature may increase the computational burden, but the diagnosis accuracy has been significantly improved compared with the previous ones.

## 7. Conclusions

In this paper, a feature extraction method based on MIGA-VMD is proposed, and the effectiveness and superiority of this method are verified by experiments. Finally, the following conclusions are drawn:

1. Taking the average value of local minimum $E_{e}$ and local minimum $R_{e}$ as the fitness function, the most suitable parameters K and α can be found by the MIGA algorithm.
2. Compared with the CEEMDAN algorithm and traditional VMD algorithm, the MIGA-VMD algorithm has obvious advantages in fault feature extraction of bearing vibration signals.
3. This feature extraction method can effectively suppress the noise interference, accurately extract the fault information of the rolling bearing vibration signal, and the accuracy of bearing fault diagnosis is effectively improved.

## Figures and Tables

**Table 1:** Rolling bearing parameters.

Model	Pitch Diameter	BallDiameter	Number of Rollers	Contact Angle
6205-2RSJEM SKF deep groove ball bearing	D(mm) 39.0398	d(mm) 7.94004	Z 9	α 0

**Table 2:** Comparison of fault diagnosis accuracy.

Literature	Feature	Classifier	Accuracy
[13]	EMD-SVD	DBN	93.55%
This Work	MIGA-VMD-SVD	DBN	95.1%
[26]	EEMD-Renyi entropy-PCA	PNN	91.7%
This Work	MIGA-VMD-Renyi entropy-PCA	PNN	93.9%
[32]	\	1-DCNN	99.34%
This Work	MIGA-VMD	1-DCNN	99.7%

## Data Availability

Data sharing not applicable.
